# Complete Genome Sequence of a Cyanotroph, *Pseudomonas fluorescens* NCIMB 11764, Employing Single-Molecule Real-Time Technology

**DOI:** 10.1128/genomeA.01111-15

**Published:** 2015-10-01

**Authors:** Lauren B. Jones, Daniel A. Kunz

**Affiliations:** Department of Biological Sciences, Division of Biochemistry and Molecular Biology, University of North Texas, Denton, Texas, USA

## Abstract

We report here the application of single-molecule real-time sequencing for determining the entire genome structure of the cyanotroph *Pseudomonas fluorescens* NCIMB 11764.

## GENOME ANNOUNCEMENT

*Pseudomonas fluorescens* NCIMB 11764 exhibits the unique ability to grow when supplied cyanide as the sole nitrogen source (cyanotrophy) (1, 2). The draft genome of this organism was previously disclosed (3), but in order to extend the analysis of genes possibly involved in cyanotrophy, the complete genome of *P. fluorescens* NCIMB 11764 was required. The draft genome sequence was assembled from Illumina GAIIX sequencing of paired-end reads into 145 contigs containing unambiguous bases using the *P. fluorescens* PF0-1 genome as a reference (4). However, to arrive at a more detailed structure of the genome and close gaps in the sequence imposed by Illumina-based sequencing technology available at the time, we sought to determine the complete nucleotide sequence. To advance from the pseudo- to the complete chromosome structure, we employed the newly developed Pacific Biosciences single-molecule real-time (SMRT) sequencing technology (5), which has the advantage over Illumina technology in yielding long reads. Here, we report the successful determination of the sequence for a single long read (contig) representing the entire *P. fluorescens* NCIMB 11764 chromosome.

*P. fluorescens* NCIMB 11764 genomic DNA was isolated using the UltraClean microbial DNA isolation kit (Mo Bio, Carlsbad, CA, USA), according to the manufacturer’s instructions. DNA was sequenced by the University of Michigan DNA Sequencing Core (Ann Arbor, MI, USA) using the PacBio *RS* II SMRT sequencing platform with P5-C3 chemistry (5). From a 10-kb insert library, 163,784 reads were generated, with an average read length of 5,341 bp across three SMRT cells. The genome was assembled employing PacBio SMRT Analysis, version 2.3.0, using the *de novo* RS_HGAP_Assembly.3 protocol, in which the hierarchical genome assembly process (HGAP) constructed a consensus sequence, and Quiver polished the final construct with 99.98% consensus concordance (6). The genome was assembled into one complete 6,998,154-bp contig having a G+C content of 59.0%. The NCBI Prokaryotic Genome Annotation Pipeline was used to predict and annotate *P. fluorescens* NCIMB 11764 genes (7), which consist of 6,365 protein- and 91 RNA-coding genes. Accordingly, the complete chromosomal structure was determined *de novo*, with genes not included in the previously described draft genome (3) now being identified, and complex sequences were further annotated. Thus, SMRT sequencing was able to produce a high-quality finished genome sequence for *P. fluorescens* NCIMB 11764, the availability of which will further facilitate genome comparisons and the identification of genes unique to cyanotrophy.

### Nucleotide sequence accession number. 

The complete *P. fluorescens* NCIMB 11764 genome sequence has been deposited in DDBJ/ENA/GenBank under the accession no. CP010945. The version described in this paper is the first version of the complete genome.
